# A structural com­parison of salt forms of dopamine with the structures of other phenyl­ethyl­amines

**DOI:** 10.1107/S2053229623007696

**Published:** 2023-09-11

**Authors:** Alan R. Kennedy, Laura Cruickshank, Pamela Maher, Zoe McKinnon

**Affiliations:** a Department of Pure & Applied Chemistry, University of Strathclyde, Glasgow, G1 1XL, United Kingdom; University of Oxford, United Kingdom

**Keywords:** crystal structure, active pharmaceutical ingredient, API, salt selection, form selection, dopamine, phenyl­ethyl­amine

## Abstract

The structures of four salt forms of dopamine are reported. These are dopamine [2-(3,4-di­hydroxy­phen­yl)ethan-1-aminium] benzoate, C_8_H_12_NO_2_^+^·C_7_H_5_O_2_^−^, **I**, dopamine 4-nitro­benzoate, C_8_H_12_NO_2_^+^·C_7_H_4_NO_4_^−^, **II**, dopamine ethane­di­sul­fon­ate, 2C_8_H_12_NO_2_^+^·C_2_H_4_O_6_S_2_^2−^, **III**, and dopamine 4-hy­droxy­benzene­sul­fon­ate monohydrate, C_8_H_12_NO_2_^+^·C_6_H_5_O_4_S^−^·H_2_O, **IV**. In all four structures, the dopamine cation adopts an extended conformation. Inter­molecular inter­action motifs that are common in the salt forms of tyramine can be found in related dopamine structures, but hydrogen bonding in the dopamine structures appear to be more variable and less predictable than for tyramine. Packing analysis discovered three dopamine-containing groups of structures that can be described as isostructural with regards to the cation positions. Two of these groups contain both dopamine and tyramine species, and one of these is also highly variable in other ways too, containing anhydrous and hydrated forms, different anion types and ionized and neutral phenyl­ethyl­amine species. As such, the group illustrates that packing behaviour can be robust and similar even where inter­molecular inter­actions such as hydrogen bonds are very different.

## Abstract 2

In four new dopamine salts, the dopamine cation adopts an extended conformation. Inter­molecular inter­action motifs that are common in the salt forms of tyramine can be found in related dopamine structures, but hydrogen bonding in the dopamine structures appear to be more variable and less predictable than for tyramine.

## Introduction

The generation of salt forms of an active pharmaceutical ingredient (API) is a well-known process used by the pharmaceutical industry to change important material properties of the API. Idealizing properties such as solubility, stability or hygroscopicity is important to the development of an effective and commercially successful API (Stahl & Wermuth, 2008 ▸). It is generally accepted that there are links between the solid-state structure and the material properties of inter­est, and that a greater understanding of such structure-to-property correlations should help to rationalize salt screening and other form-choice processes.

Phenyl­ethyl­amine (PEA) com­pounds (Scheme 1) have long been known to have a large variety of pharmaceutical and bio­logical roles (*e.g.* Brown *et al.*, 1979 ▸; Drew *et al.*, 1978 ▸; Broadley, 2010 ▸; Dennany *et al.*, 2015 ▸). Due to their favourable handling characteristics, several have been used in studies where relatively large numbers of salt forms of a given API have been crystallographically characterized and the structures then used to systematically investigate material properties. The earliest examples of this are the studies by Davey investigating the structures and crystal properties of pseudo­ephedrine salts forms and their relationships to solubility (Black *et al.*, 2007 ▸; Collier *et al.*, 2006 ▸). A problem with similar studies using large numbers of crystal structures is how to simply com­pare and contrast multiple structures. One solution to this are the packing similarity tools available within *Mercury* (Taylor & Wood, 2019 ▸; Childs *et al.*, 2009 ▸; Macrae *et al.*, 2020 ▸). In the area of PEA salt forms, these tools have been used to investigate hydrate formation in tyramine salt forms (Briggs *et al.*, 2012 ▸), and density and melting point in pairs of enanti­opure and racemic methyl­ephedrine salt forms (Kennedy *et al.*, 2011 ▸). An intriguing result using this approach was that groups of methyl­ephedrine salt forms that showed isostructural cation packing also showed tighter correlation between aqueous solubility and melting point than did similar salt forms that were not part of isostructural packing groups (de Moraes *et al.*, 2017 ▸). 
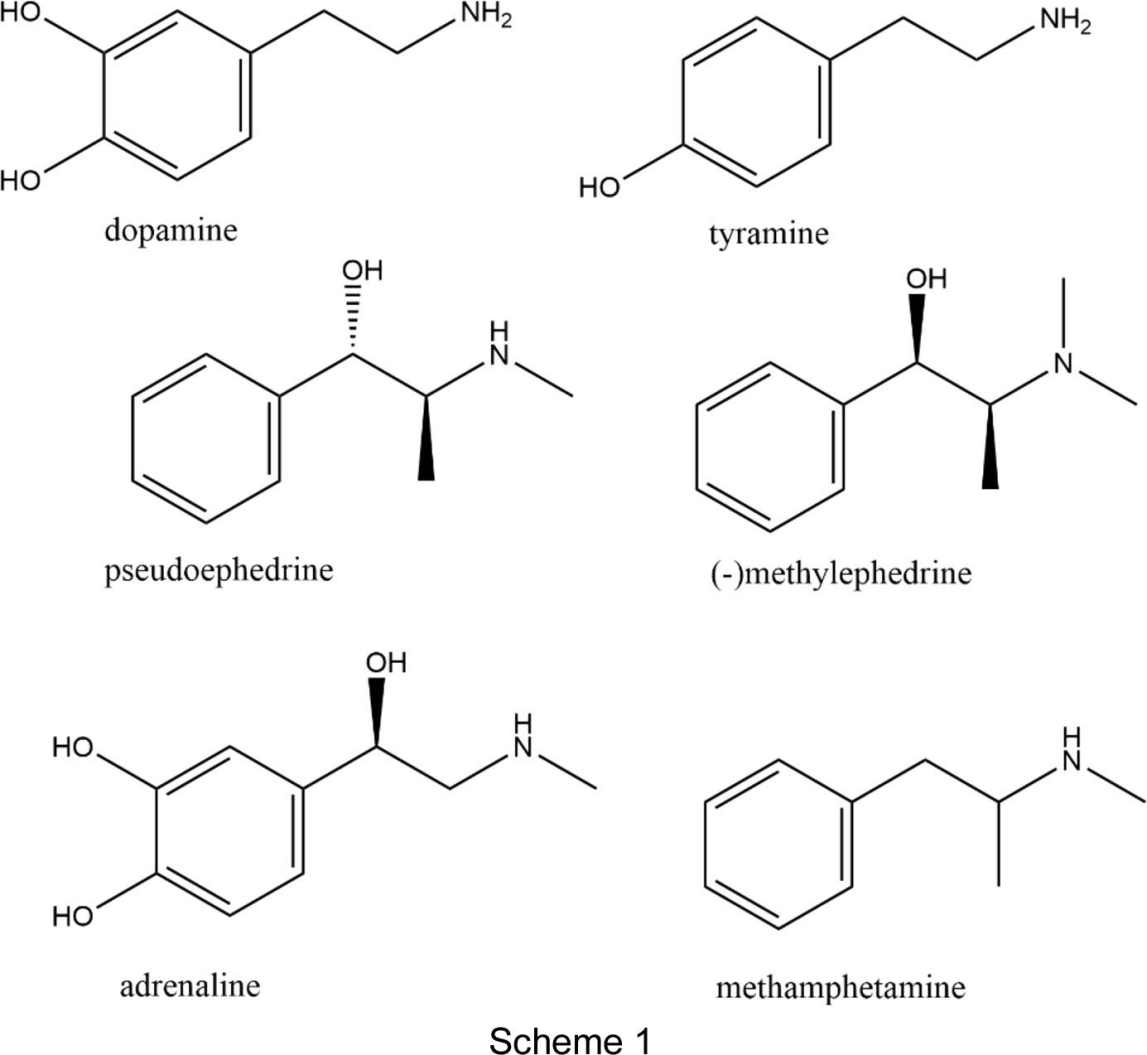


Dopamine is a biologically significant member of the PEA family and arguably the most well known. In the brain it is a neurotransmitter and it is known to play a role in a wide range of human bodily functions, including motor control, motivation, gastrointestinal tract function and operation of the im­mune system. It is well known that loss of the ability to secrete dopamine leads to Parkinson’s disease (*e.g.* Wenzel *et al.*, 2015 ▸; Schultz, 2007 ▸). Perhaps less well known is that dopamine in the form of its HCl salt is used as an API, for instance, in the treatment of neonatal shock (Noori *et al.*, 2003 ▸). Some crystallographic work has been undertaken on forms of dopamine. Dopamine itself has been shown to exist in the solid as a zwitterionic form, with deprotonation of the OH group *meta* to the ethyl­amine substituent (Cruickshank *et al.*, 2013 ▸). The structures of some simple salt forms of dopamine are also know. These include forms with inorganic anions [the halides DOPAMN01, QQQAEJ02 and ATOLUR04 (Giesecke, 1980 ▸; Pike & Dziura, 2013 ▸; Ivanova & Spiteller, 2017 ▸); the nitrate CIZYAN (Gatfaoui *et al.*, 2014 ▸); and the perchlorate OGEGAJ (Boghaei *et al.*, 2008 ▸)] and four forms with small to medium sized organic anions (ATOLIF, ATOMAY, RAWDEB and MIYLOV; Ivanova & Spiteller, 2010 ▸; Feng *et al.*, 2017 ▸; Ohba & Ito, 2002 ▸). This gives a total of ten relevant literature structures available from the Cambridge Structural Database (CSD, Version 5.43, update of November 2022, Groom *et al.*, 2016 ▸). To this data set we herein add the structures of the benzoate, 4-nitro­benzoate, ethane­disulfonate and 4-hy­droxy­benzene­sulfonate salt forms of dopamine. The new structures are described and a com­parative analysis of the packing of dopamine and its salt forms, and those of the closely related PEA species tyramine is presented.

## Experimental

### Synthesis and crystallization

Dopamine hydro­chloride was purchased from Sigma–Aldrich. Because of the well-known rapid oxidation of dopamine under basic conditions (Richter & Waddell, 1983 ▸), the HCl salt was converted to neutral dopamine under an N_2_ at­mo­sphere and in a Schlenk tube. This was done by addition of NaOH to ice-cooled aqueous solutions of dopamine HCl. Dopamine free base precipitated in 51–59% yield after 2 h. The white solid was separated by filtration and stored under N_2_ before use. Salt forms **I** to **IV** were prepared by adding dopamine (0.2 g) and an equimolar amount of the appropriate acid to degassed water (5 ml). The mixtures were stirred and heated under N_2_ to 313 K before being filtered to leave clear solutions. These solutions were left to evaporate slowly. Crystals suitable for single-crystal diffraction were obtained directly from these solutions within one week. For both **I** and **II**, crystals of the salt form grew alongside a small number of crystals of the parent benzoic acid.

### Refinement

The anion of **IV** was found to be disordered, with the benzene ring rotated by approximately 52° around an axis that runs through atoms S1, C9, C12 and O6. Thus, four aromatic C—H groups were each modelled as split over two sites with occupancies refined to a 50:50 ratio. No further restraints or constraints were required to satisfactorily model these disordered atoms. For **I**, **II** and **III**, all H atoms bonded to O or N atoms were positioned as found by difference synthesis and refined freely and isotropically. For **IV**, restraints on the O—H bond lengths were required and they were set to 0.88 (1) Å. For **I** to **IV**, H atoms bound to C atoms were placed in idealized positions and refined in riding modes. C—H bond lengths of 0.95 and 0.99 Å were used for CH and CH_2_ groups, respectively, and *U*_iso_(H) values were set at 1.2*U*_eq_(C) of the parent atom. Further crystallographic details and refinement parameters are given in Table 1 ▸.

## Results and discussion

**Figure 1:**
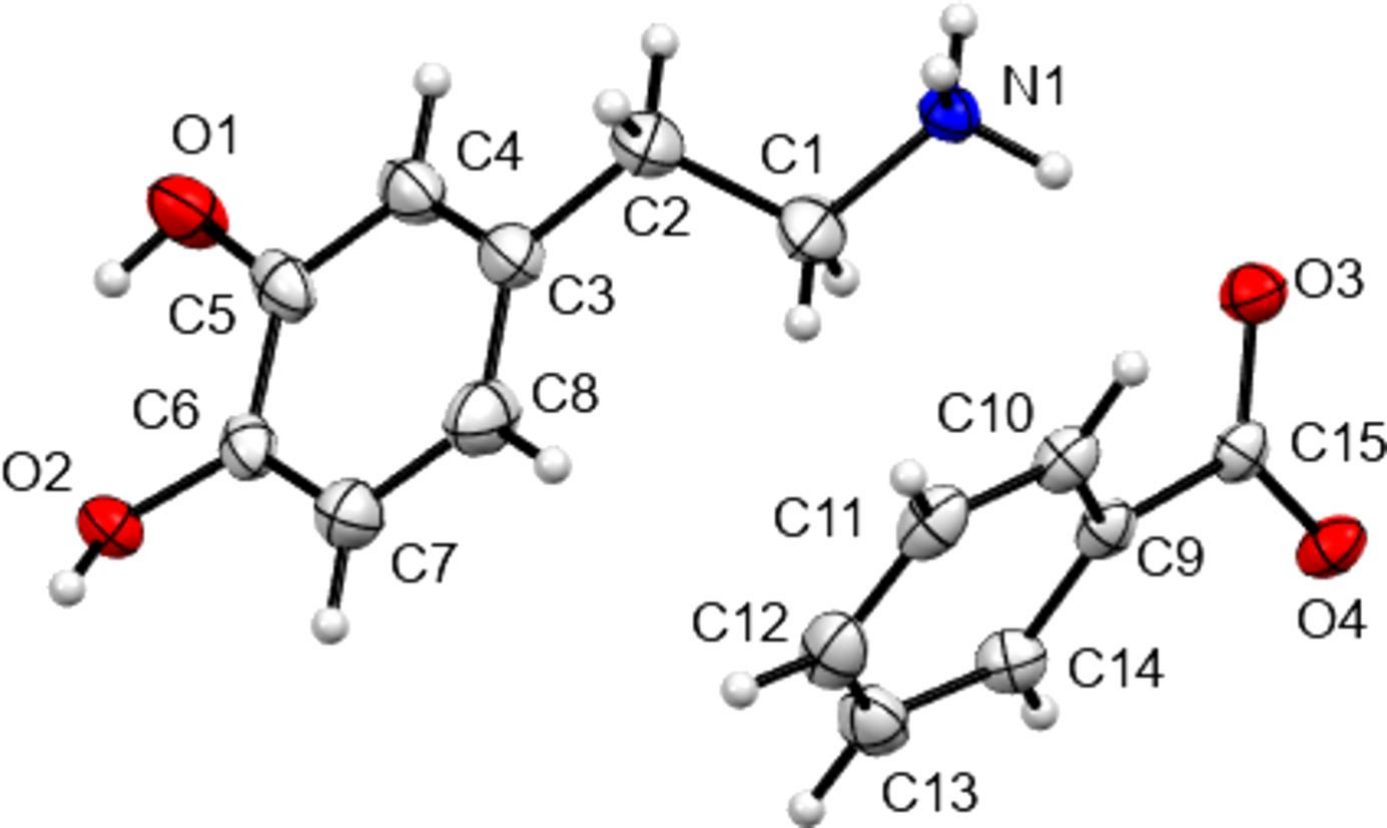
View of the asymmetric unit contents of **I**, with non-H atoms shown as 50% probability displacement ellipsoids. Here and in Figs. 2 ▸–4 ▸ ▸, H atoms are drawn as spheres of arbitrary size.

The structures of **I**–**IV** are shown in Figs. 1 ▸–4 ▸ ▸ ▸, with crystallographic parameters detailed in Table 1 ▸ and hydrogen-bonding parameters detailed in Tables 2 ▸–5 ▸ ▸ ▸. The asymmetric units of both **I** and **II** consist of a dopamine cation and a (substituted) benzoate anion. The asymmetric unit of **III** consists of a dopamine cation and half of an ethane­disulfonate dianion. Here the dianion has a crystallographic centre of symmetry in the middle of its C—C bond. Finally, **IV** is a monohydrate and so the asymmetric unit consists of a dopamine cation, a disordered hy­droxy­benzene­sulfonate anion and a water mol­ecule. In all four cases, the dopamine moiety has been protonated at the amine group and the H atom of the *meta*-hy­droxy substituent is orientated towards the O atom of the *para*-hy­droxy group to form an intra­molecular hydrogen bond. All of the ethyl­ammonium chains adopt extended con­formations, with the N1—C1—C2—C3 torsion angles ranging from −168.89 (14) to 176.39 (16)°. This corresponds to an *anti* arrangement of the large aromatic and NH_3_ substituents on the C1—C2 fragment. The relevant literature salt forms as listed in Table 6 ▸ also adopt extended conformations, with the exception of the di­nitro­benzoate salt MIYLOV. This is the only form to have a folded conformation, displaying an N—C—C—C torsion angle of 60.5° (Ohba & Ito, 2002 ▸). This distribution of conformations resembles that found for the salt forms of the closely related tyramine cation. Of 42 tyramine salt forms, the majority displayed extended conformations and only four displayed a folded conformation (Briggs *et al.*, 2012 ▸).

The dopamine cations of **I**–**IV** utilize all three NH groups and both OH groups as hydrogen-bond donors, but apart from that, there is little similarity between them and in detail each acts in a different manner. As shown in Table 7 ▸, in benzoate **I** all potential donors make a single hydrogen bond to an O atom of a benzoate COO group. Here, none of the atoms of the cation acts as an acceptor and hydrogen bonds exist only between cations and anions. In **II**, the number of potential hydrogen-bond acceptors is increased by the inclusion of an NO_2_ group on the anion. This leads to the cation making extra donor inter­actions, with one NH group and the *para*-OH group both acting as bifurcated donors to two hydro­gen bonds. Again, no atom of the cation acts as an acceptor and all hydrogen bonds are formed between cations and anions. In **III**, all five cation donor groups make single hydrogen bonds, but in contrast to **I** and **II**, the *para*-OH group also acts as an acceptor, accepting a hydrogen-bond contact from a neighbouring *R*NH_3_ group. Thus, in **III**, there are hydrogen bonds both between cations and anions, and between pairs of cations. In the hydrate **IV**, one of the NH groups makes a bifurcated donor inter­action with two neighbouring SO_3_ groups and all other cation donor atoms make single hydrogen bonds. That from the *para*-OH group donates to a water mol­ecule, but all others are to anions. The *para*-OH group also accepts a hydrogen bond from a water mol­ecule. In **IV**, hydrogen bonds link cations to anions, and both cations and anions to water. However, unlike **III**, there are no cation-to-cation hydrogen-bond contacts.

**Figure 5:**
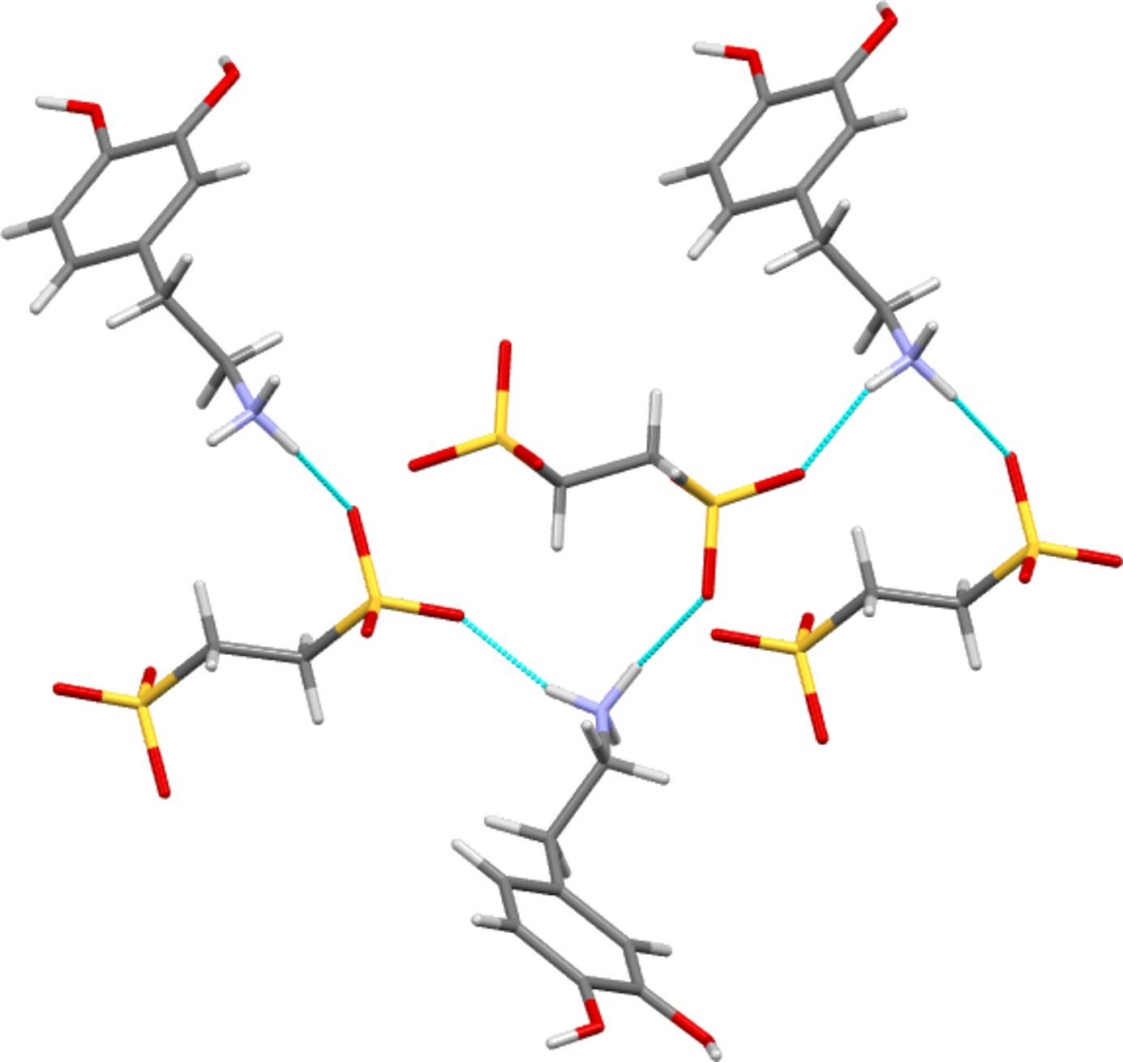
Part of the 1D 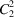 (6) motif found in **III**. The hydrogen-bonded chain propagates parallel to the *a* direction.

**Figure 7:**
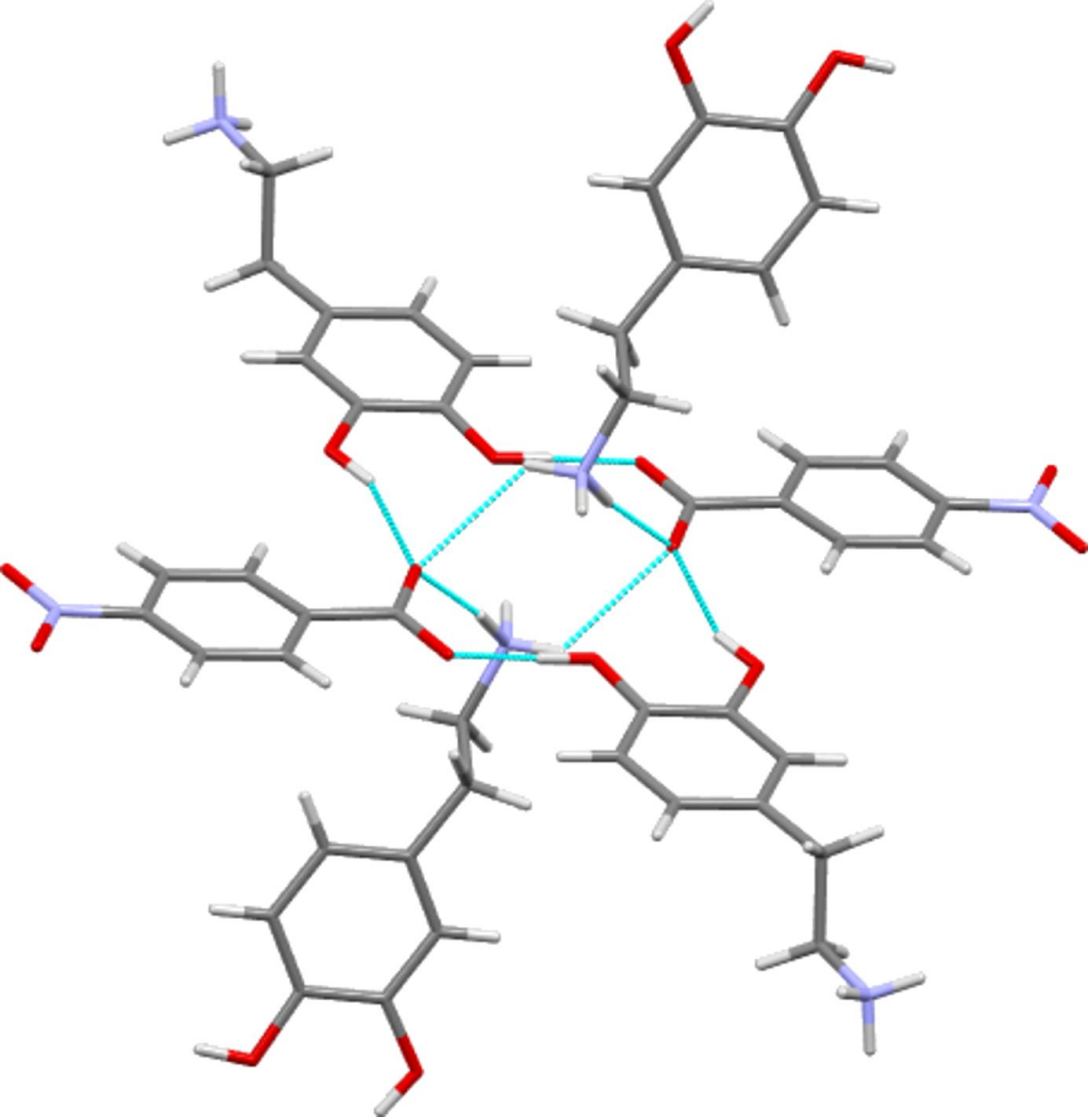
A central hydrogen-bonding motif in **II** consisting of four coplanar groups linked *via* the catechol and carboxyl­ate groups. This unit is capped top and bottom by hydrogen bonds to the *R*NH_3_ group of the cation.

A large-scale structural study on tyramine salt forms identified two common hydrogen-bonding motifs that co-existed in 19 of 24 benzoate and sulfonate salts of that com­pound (Briggs *et al.*, 2012 ▸). These motifs were both one-dimensional (1D) chain structures, one of graph set 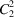 (6) corresponding to an (⋯O*X*O⋯HNH⋯)_*n*_ (*X* = C or S) linkage and one of graph set 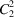 (13) corresponding to COO or SO_3_ groups bonding to both the NH_3_ head and the *para*-OH tail of the cation. Here only structures **I** and **III** show both motifs. They also have an additional 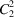 (12) motif. This latter is equivalent to the 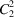 (13) motif, but utilizes the extra *meta*-OH group of dopamine rather than the *para*-OH group which is common to both dopamine and tyramine. Figs. 5 ▸ and 6 ▸ illustrate these hydro­gen-bonded-chain features. Structure **IV** contains both the 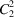 (6) and the 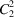 (12) motifs, but the *para*-OH group of this hydrate only hydrogen bonds to water mol­ecules and thus the 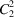 (13) motif does not occur here. In contrast, of the three motifs described above, the nitro­benzoate salt **II** only displays the 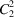 (13) chain. In the hydrogen bonding of structure **II**, ring motifs become prevalent, including that formed by a tetra­mer consisting of two cations and two anions. These are linked by hydrogen bonding between the catechol moieties and the carboxyl­ate groups in an 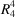 (18) motif. This coplanar tetra­mer is then capped on each side of the plane through hydrogen bonds to the *R*NH_3_ groups of two further cations, as shown in Fig. 7 ▸.

**Figure 8:**
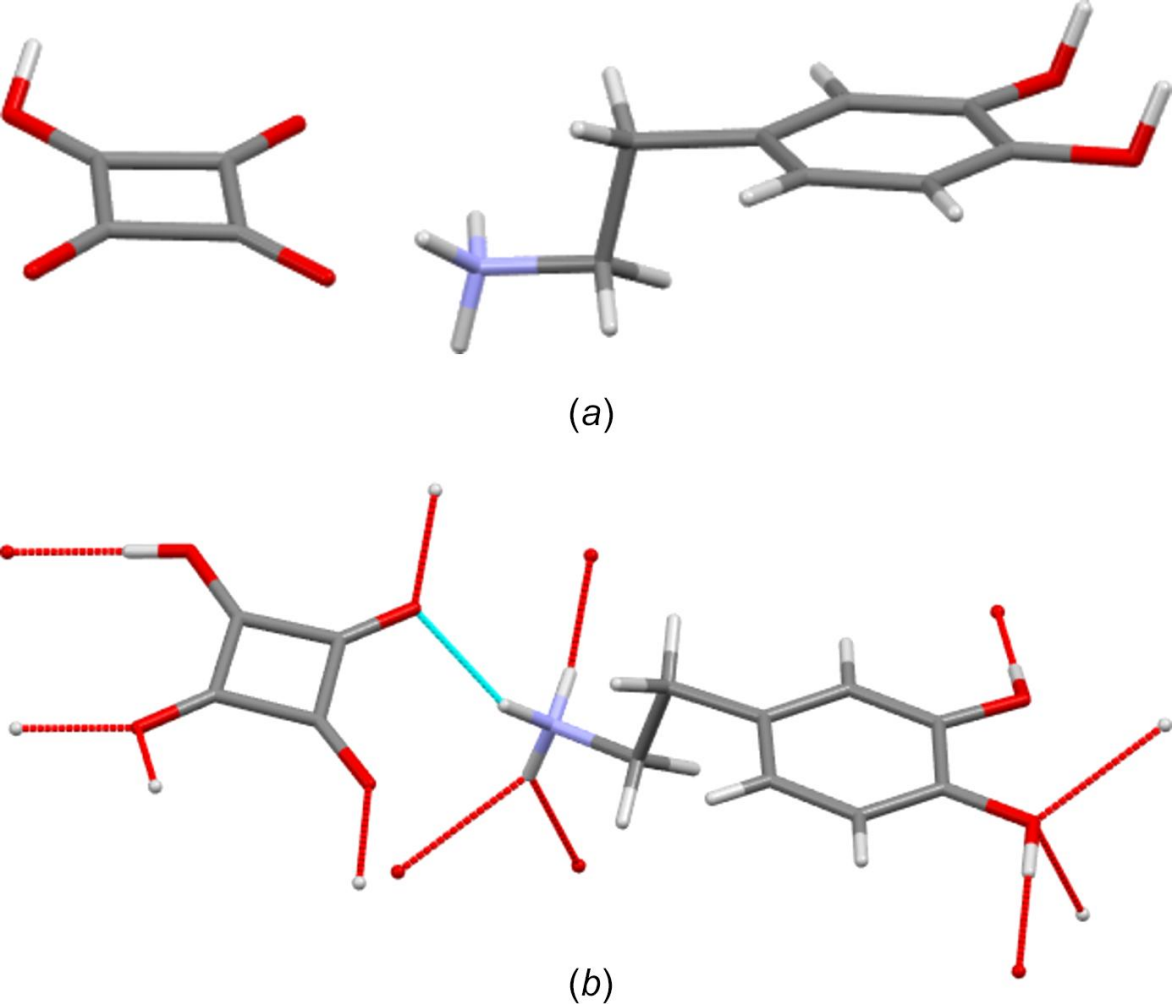
(*a*) The structure of ATOMAY as recovered from the CSD. Note the unusual out-of-plane geometry of the H atoms of the OH substituents. These out-of-plane H atoms do not form hydrogen bonds with neighbouring ions. (*b*) The structure of ATOMAY edited so as to place H atoms in-plane and in geometries that maximize hydrogen bonding.

On attempting to com­pare the hydrogen-bonding behaviour of dopamine cations in structures **I**–**IV** with that in the literature salt forms, a problem was observed. The H-atom positions recovered for the structures of ATOLIF, ATOMAY and, to a certain extent, ATOLUR04 appeared unusual (Fig. 8 ▸) (Ivanova & Spiteller, 2010 ▸, 2017 ▸). As well as unusual out-of-plane conformations, those groups which would be expected to be strong hydrogen-bond donors did not connect with geometrically reasonable acceptor atoms, in contradiction to Etter’s rules (Etter, 1990 ▸). In all three cases, alternative H-atom positions that did give typical hydrogen-bond inter­actions were available. Thus, for these structures, before hydrogen-bonding motifs were analysed, the H atoms were removed and manually replaced with the H atoms situated so as to give typical hydrogen-bonding geometries. For the hydrate ATOLUR04, H atoms were missing from the water mol­ecule sites. Thus, before analysis, these H atoms were also added in geometrically reasonable expected positions. All further discussion of the hydrogen bonding of these three com­­pounds thus refers to the edited structures. Atomic structures for these edited structures are given in CIF format in the supporting information.

When examining all 13 available salt forms of dopamine in Table 7 ▸, it becomes apparent that the hydrogen-bonding be­haviour of the cation is very variable. Of 14 crystallo­graphically independent dopamine fragments, only two, those of the chloride DOPAMN01 and the bromide QQQEAJ01, have the same set of inter­actions originating from the dopamine cation. Furthermore, none of the literature carboxyl­ate or sulfonate structures feature the combination of 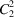 (6) and 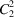 (13) chains that is found to be prevalent in tyramine salt forms (Briggs *et al.*, 2012 ▸). The halide salt forms of dopamine (Cl, Br and I) do though present the 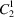 (4), 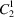 (11) and 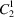 (10) chains that are the monoatomic ion equivalent of the three motifs discussed above for COO- and SO_3_-based ions. Finally, it is noted that the ladder-like structures commonly seen for other carboxyl­ate salt forms of *R*NH_3_^+^ species (Kinbara *et al.*, 1996 ▸) are found for neither dopamine salt forms nor for tyramine salt forms.

**Figure 9:**
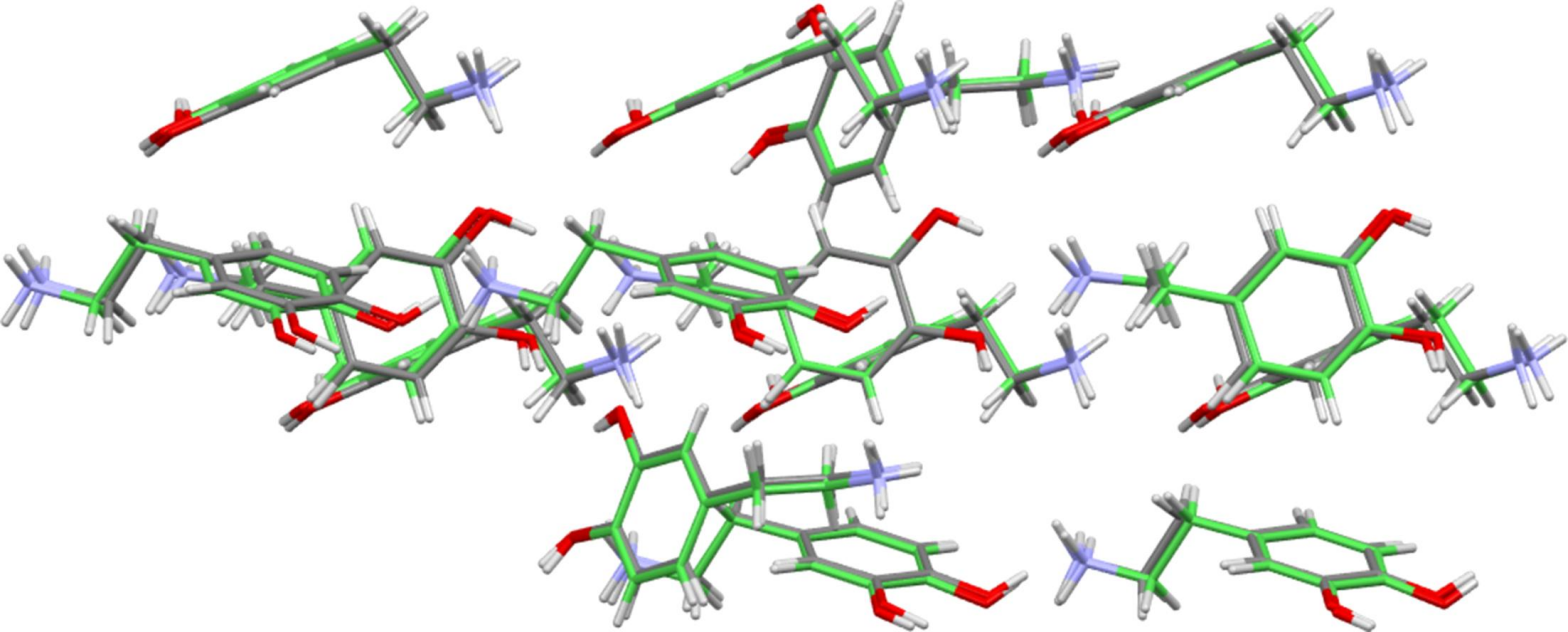
Overlay diagram showing the packing of 15 dopamine cations of the chloride structure (multicoloured) and 15 dopamine cations of the bromide structure (green). The r.m.s. value is 0.210 Å. Anions have been omitted for clarity.

In an attempt to find further com­parable features across the structural group, use was made of the ‘crystal packing similarity’ module within *Mercury* (Macrae *et al.*, 2020 ▸; Childs *et al.*, 2009 ▸). This was used to investigate similarity in the cation packing across the available dopamine forms by investigating geometrical similarity between small clusters of dopamine cations. In doing so other species present, such as anions and solvent mol­ecules, were ignored. An initial calculation using only the dopamine structures identified that the chloride and bromide salts of dopamine were isostructural at a cluster size of 15 cations, and that the iodide and perchlorate salt forms were similarly isostructural (Figs. 9 ▸ and 10 ▸). For the Cl/Br pair, the reported unit cells and space groups clearly indicate that the com­plete structures are both isostructural and isomorphous (Giesecke, 1980 ▸; Pike & Dziura, 2013 ▸). However, there is no such similarity in the unit cells of the I/ClO_4_ pair (Ivanova & Spiteller, 2017 ▸; Boghaei *et al.*, 2008 ▸).

**Figure 11:**
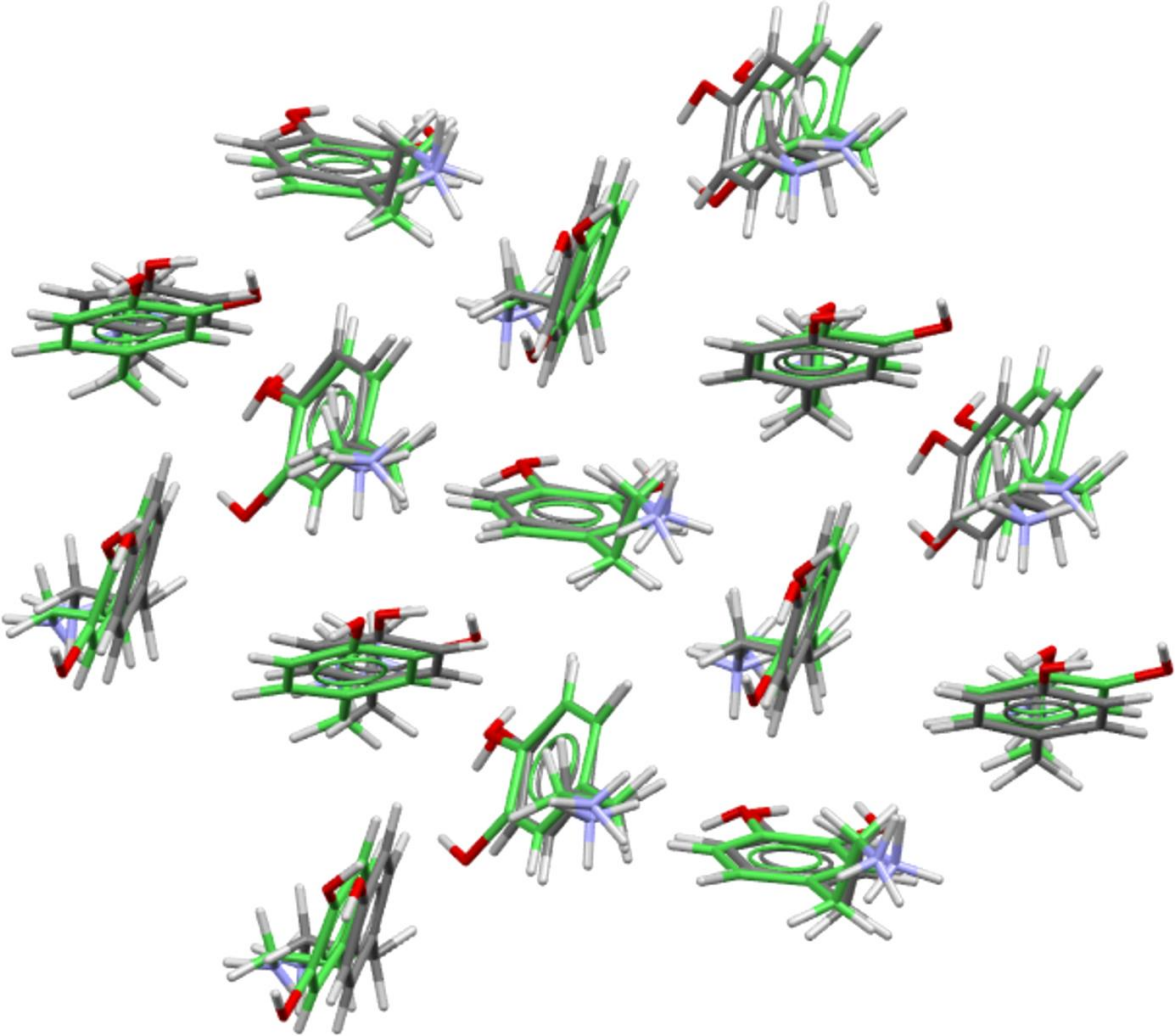
Overlay diagram showing the packing of 15 dopamine cations of the chloride structure (green) and 15 tyramine units of the hemihydrate structure. The r.m.s. value is 0.566 Å. Anions and water mol­ecules have been omitted for clarity.

More inter­esting results were obtained when the packing similarity module was applied to a data set that included both the available dopamine forms and those of tyramine. This time six groups of structures with similar cation packing arrays at the level of a 15 from 15 match were identified (Table 8 ▸). Group 1 contains only the dopamine I/ClO_4_ pair as already seen, and groups 4, 5 and 6 contain only tyramine structures. However, groups 2 and 3 are inter­esting as they contain both dopamine and tyramine structures. Group 2 contains **I**, the benzoate salt of dopamine, and both the 4-amino- and 4-methyl­benzoate salt forms of tyramine. Group 3 is an expanded version of the dopamine Cl/Br grouping that now also contains **III**, the ethane­disulfonate salt of dopamine, and six forms of tyramine. These are the chloride, bromide, perchlorate, BF_4_ and di­hydrogen phosphate salts of tyramine, and also the neutral hemihydrate of tyramine. (Note: when assessing dopamine structures alone, the ethane­disulfonate structure **III** was found to be related to the Cl/Br group, but only at a level with 7 from 15 matches. However, the larger and more varied dopamine/tyramine group appears to allow a fuller match.) Each group of Table 8 ▸ is com­posed of structures with broadly similar anion types. Thus, for example, group 2 contains only structures with benzoate or *para*-benzoate anions, and group 3 is com­posed of species with simple inorganic anions or coformers. Beyond this basic similarity though lies a great deal of variation. For example, group 3 encom­passes structures with different cations, with different anions, with and without solvent present, and with neutral PEA species rather than charged ones, see Fig. 11 ▸ as an example.

This, of course, leads to very different hydrogen bonding throughout the structures of these com­pounds. From a chemical identity point of view, the two most different structures of group 3 are the ethane­disulfonate salt of dopamine, **III**, and the hemihydrate of tyramine, TIRZEB (Cruickshank *et al.*, 2013 ▸). The ethane­disulfonate dianion does not fit well with the descriptor used above of a ‘simple inorganic anion’ being as it is both larger than the other anions in this group and doubly charged. This is reconciled simply. The ethane­disulfonate ion lies upon a crystallographic centre of sym­metry, thus making the unique repeating structural part the much smaller ‘O_3_SCH_2_’ fragment which is a reasonable match to a ‘simple inorganic anion’. The ethane­disulfonate anion takes the structural place of two anions in other group member structures, such as that of dopamine chloride, DOPAMN01. The intra­molecular S⋯S separation of 4.338 Å in **III** com­pares well with the inter­molecular Cl⋯Cl distance of 4.303 Å in the chloride. Tyramine hemihydrate, TIRZEB, is an inter­esting structure. Disorder of the phenol H-atom positions in this structure means that the tyramine fragments present are best thought of as a mix of neutral, cationic and zwitterionic forms of tyramine (Cruickshank *et al.*, 2013 ▸). This presents some difference to the cationic PEA forms that make up the rest of group 3. Water is obviously a neutral coformer rather than the small anions found in the rest of group 3, but a further difference is stoichiometry. There is only one water mol­ecule per two tyramine fragments in TIRZEB, as opposed to one monoanionic fragment per organic cation in all the other structures of group 3. Despite all this variation in chemical identity and in the type and number of the strong hydrogen-bonding inter­molecular inter­actions, the cations of these groups still adopt similar packing arrangements. Con­versely, similarities in hydrogen bonding do not necessarily seem to lead to similarity in cation packing. One of the few patterns in the hydrogen-bonding behaviour of the dopamine salts is that all three halides have structures based around the same chain-type inter­actions (see above). Despite this hydro­gen-bonding similarity, only the Cl and Br salt forms are found in group 3, with the iodide salt grouping with the perchlorate in group 1. A similar observation that PEA cation packing was not governed by hydrogen-bond formation, though one from a data set that did not feature different cations, was made in a study on methyl­ephedrine salt forms (Kennedy *et al.*, 2011 ▸). In a related point, Collier *et al.* (2006 ▸) noted that it was simply the gross amphiphilic nature of the ephedrine cation that dominated packing in structures of its salt forms, rather than the detail of the functional groups or individual inter­action types.

## Supplementary Material

Crystal structure: contains datablock(s) I, II, III, IV, global. DOI: 10.1107/S2053229623007696/op3027sup1.cif

Structure factors: contains datablock(s) I. DOI: 10.1107/S2053229623007696/op3027Isup2.hkl

Supporting information file. DOI: 10.1107/S2053229623007696/op3027Isup6.cml

Structure factors: contains datablock(s) II. DOI: 10.1107/S2053229623007696/op3027IIsup3.hkl

Supporting information file. DOI: 10.1107/S2053229623007696/op3027IIsup7.cml

Structure factors: contains datablock(s) III. DOI: 10.1107/S2053229623007696/op3027IIIsup4.hkl

Supporting information file. DOI: 10.1107/S2053229623007696/op3027IIIsup8.cml

Structure factors: contains datablock(s) IV. DOI: 10.1107/S2053229623007696/op3027IVsup5.hkl

Supporting information file. DOI: 10.1107/S2053229623007696/op3027IVsup9.cml

CCDC references: 2292822, 2043930, 2292821, 2043931

## Figures and Tables

**Figure 2:**
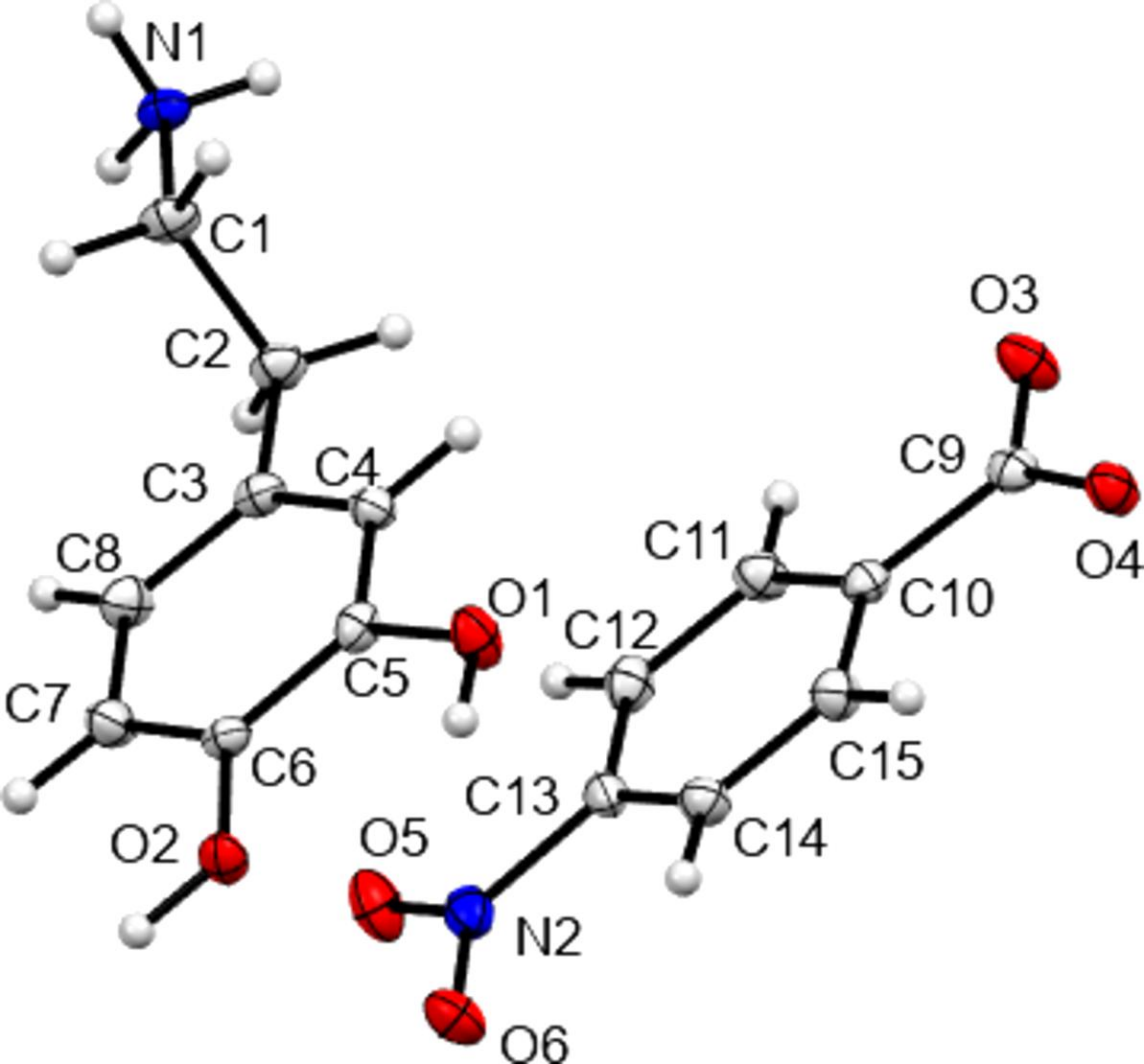
View of the asymmetric unit contents of **II**, with non-H atoms shown as 50% probability displacement ellipsoids.

**Figure 3:**
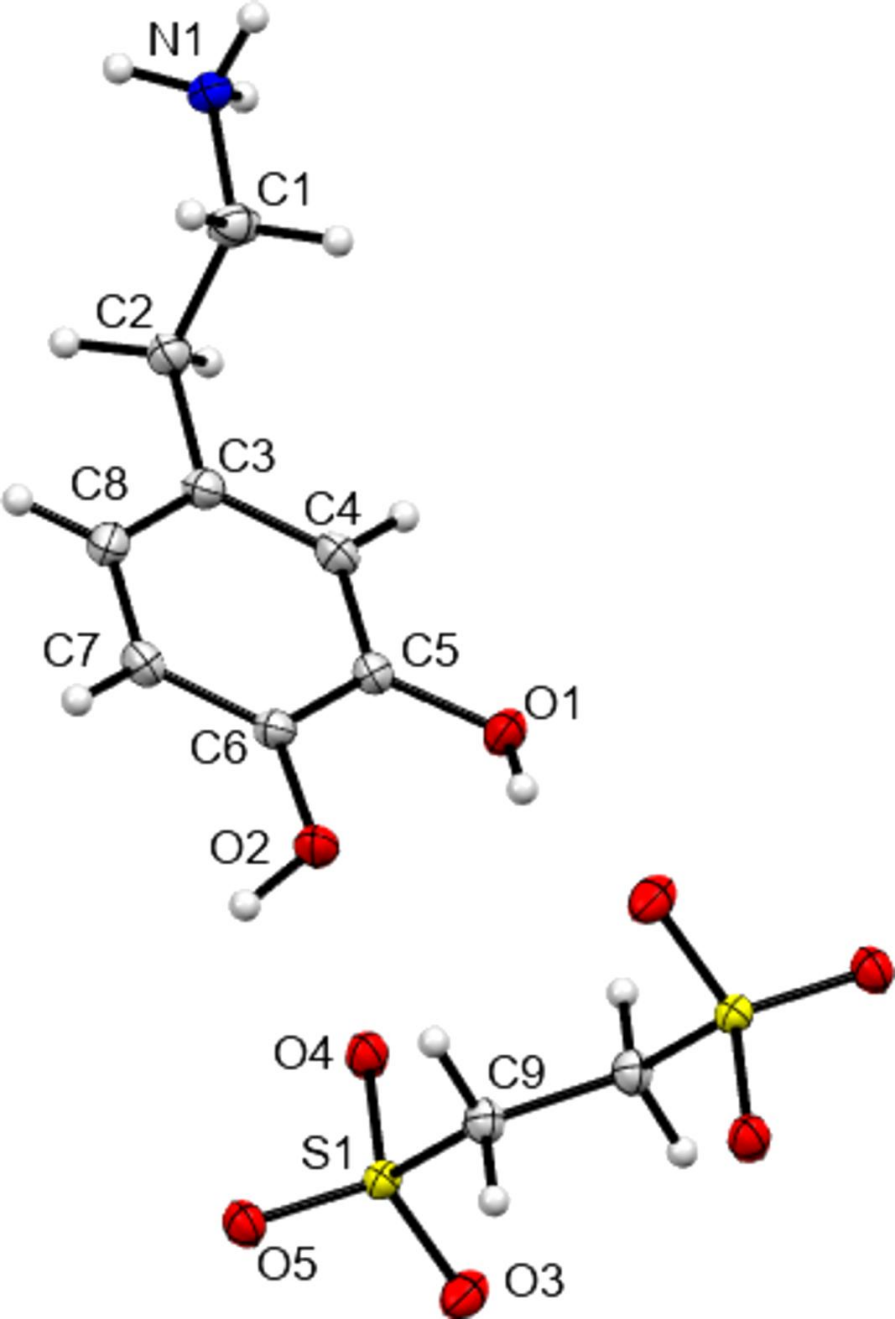
View of the asymmetric unit contents of **III**, extended to show the com­plete dianion generated by the inversion centre in the C—C bond. Non-H atoms are shown as 50% probability displacement ellipsoids.

**Figure 4:**
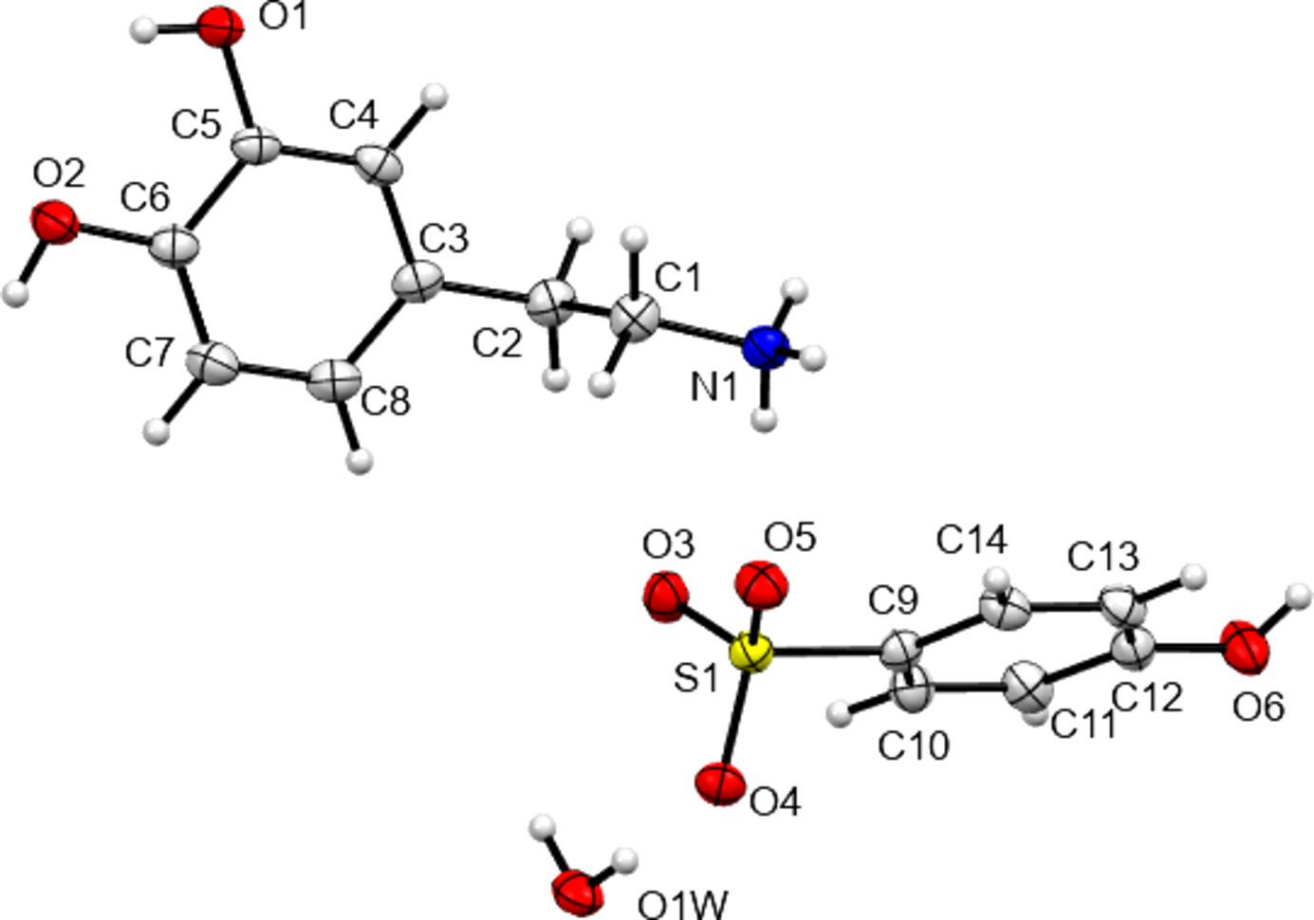
View of the asymmetric unit contents of **IV**, with disorder of atoms C10, C11, C13 and C14 hidden for clarity. Non-H atoms are shown as 50% probability displacement ellipsoids.

**Figure 6:**
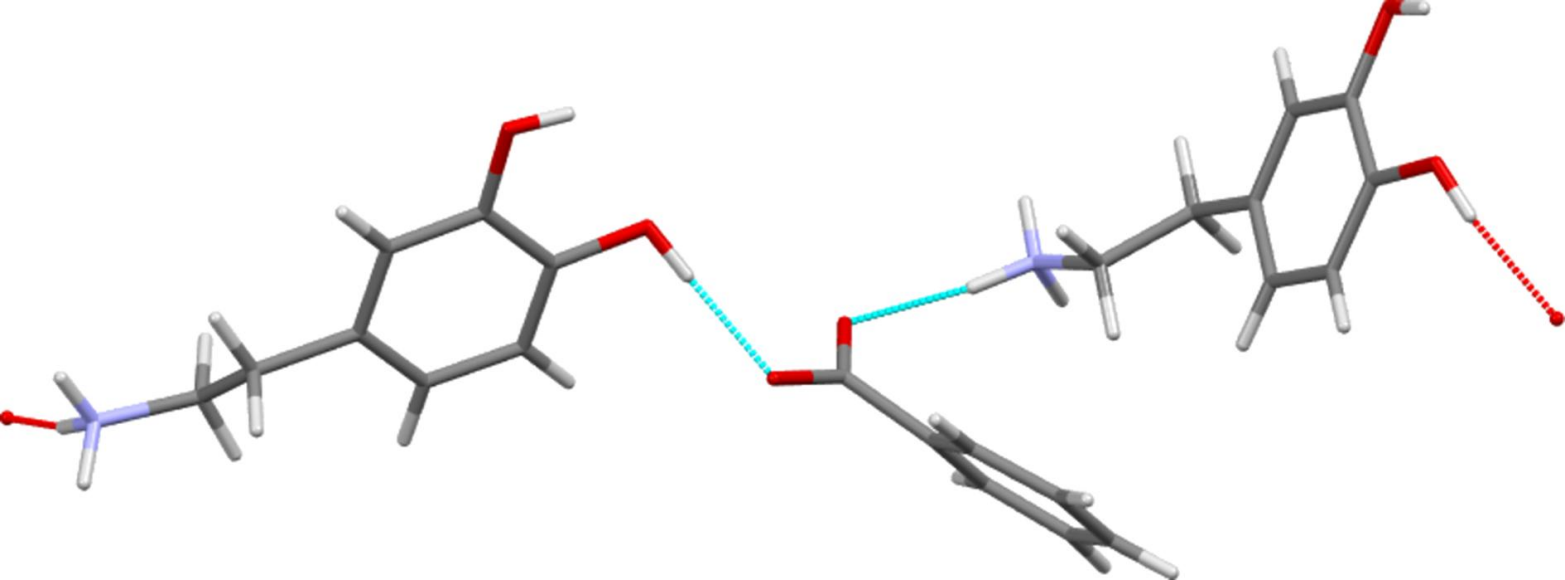
An illustration showing the repeating core of the 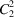 (13) motif, utilizing the *para*-OH group, of structure **I**. A similar 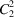 (12) motif, using the same functional groups but utilizing the *meta*-OH group rather than the *para*-OH group, also exists here.

**Figure 10:**
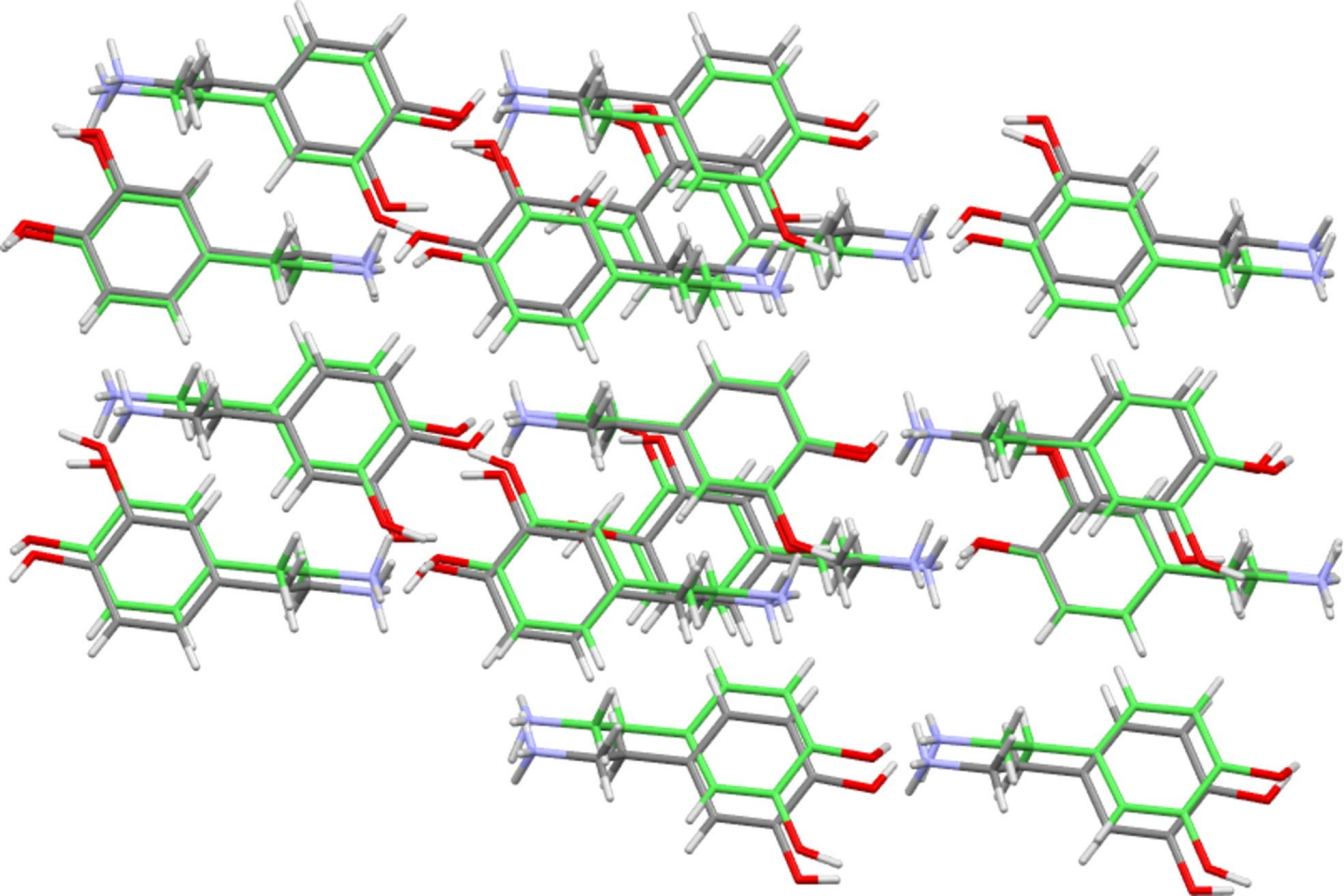
Overlay diagram showing the packing of 15 dopamine cations of the perchlorate structure (multicoloured) and 15 dopamine cations of the iodide structure (green). The r.m.s. value is 0.451 Å. Anions have been omitted for clarity.

**Table 1:** Experimental details For all structures: *Z* = 4. Experiments were carried out at 123 K. Absorption was corrected for by multi-scan methods (*CrysAlis PRO*; Rigaku OD, 2019 ▸). H atoms were treated by a mixture of independent and constrained refinement.

	**I**	**II**	**III**	**IV**
Crystal data				
Chemical formula	C_8_H_12_NO_2_^+^·C_7_H_5_O_2_^−^	C_8_H_12_NO_2_^+^·C_7_H_4_NO_4_^−^	2C_8_H_12_NO_2_^+^·C_2_H_4_O_6_S_2_^2−^	C_8_H_12_NO_2_^+^·C_6_H_5_O_4_S^−^·H_2_O
*M*_r_	275.29	320.30	496.54	345.36
Crystal system, space group	Monoclinic, *P*2_1_/*c*	Monoclinic, *P*2_1_/*n*	Orthorhombic, *P* *b* *c* *a*	Monoclinic, *P*2_1_/*c*
*a*, *b*, *c* (Å)	11.7637 (6), 11.7460 (5), 10.3316 (5)	7.4993 (2), 18.5051 (5), 10.7610 (3)	10.6328 (5), 8.5651 (5), 23.8669 (13)	17.4085 (8), 11.9234 (5), 7.6779 (3)
α, β, γ (°)	90, 111.384 (6), 90	90, 99.628 (3), 90	90, 90, 90	90, 95.273 (4), 90
*V* (Å^3^)	1329.30 (12)	1472.33 (7)	2173.6 (2)	1586.95 (12)
Radiation type	Cu *K*α	Mo *K*α	Cu *K*α	Cu *K*α
μ (mm^−1^)	0.83	0.11	2.75	2.15
Crystal size (mm)	0.35 × 0.10 × 0.02	0.35 × 0.25 × 0.05	0.30 × 0.15 × 0.05	0.5 × 0.15 × 0.07

Data collection				
Diffractometer	Oxford Diffraction Gemini S	Oxford Diffraction Xcalibur E	Oxford Diffraction Gemini S	Oxford Diffraction Gemini S
*T*_min_, *T*_max_	0.761, 1.000	0.928, 1.000	0.500, 1.000	0.653, 1.000
No. of measured, independent and observed [*I* > 2σ(*I*)] reflections	5127, 2614, 2033	7174, 3573, 2620	7583, 2172, 1924	6603, 3113, 2474
*R*_int_	0.026	0.026	0.032	0.040
(sin θ/λ)_max_ (Å^−1^)	0.619	0.682	0.626	0.621

Refinement				
*R*[*F* ^2^ > 2σ(*F* ^2^)], *wR*(*F* ^2^), *S*	0.060, 0.171, 1.02	0.048, 0.112, 1.03	0.041, 0.134, 1.14	0.057, 0.169, 1.04
No. of reflections	2614	3573	2172	3113
No. of parameters	201	228	165	277
No. of restraints	0	0	0	5
Δρ_max_, Δρ_min_ (e Å^−3^)	0.63, −0.27	0.34, −0.23	0.62, −0.57	0.81, −0.41

Computer programs: *CrysAlis PRO* (Rigaku OD, 2019 ▸), *SIR92* (Altomare *et al.*, 1994 ▸), *SHELXS* (Sheldrick, 2015*a* ▸), *SHELXL2018* (Sheldrick, 2015*b* ▸), *Mercury* (Macrae *et al.*, 2020 ▸) and *SHELXL* in *WinGX* (Farrugia, 2012 ▸).

**Table 2:** Hydrogen-bond geometry (Å, °) for **I** [Chem scheme1]

*D*—H⋯*A*	*D*—H	H⋯*A*	*D*⋯*A*	*D*—H⋯*A*
O1—H1*H*⋯O2	0.84 (4)	2.29 (4)	2.736 (3)	113 (3)
O1—H1*H*⋯O3^i^	0.84 (4)	2.17 (4)	2.872 (3)	141 (3)
O2—H2*H*⋯O4^ii^	0.85 (4)	1.83 (4)	2.666 (2)	171 (3)
N1—H1*N*⋯O4^iii^	0.98 (3)	1.87 (4)	2.834 (3)	167 (3)
N1—H2*N*⋯O3	0.95 (3)	1.93 (3)	2.867 (3)	171 (3)
N1—H3*N*⋯O3^iv^	0.87 (4)	2.04 (4)	2.866 (3)	156 (3)

Symmetry codes: (i) 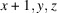 ; (ii) 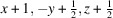 ; (iii) 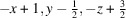 ; (iv) 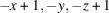 .

**Table 3:** Hydrogen-bond geometry (Å, °) for **II** [Chem scheme1]

*D*—H⋯*A*	*D*—H	H⋯*A*	*D*⋯*A*	*D*—H⋯*A*
O1—H1*H*⋯O2	0.87 (2)	2.31 (2)	2.7384 (17)	110.4 (19)
O1—H1*H*⋯O4^i^	0.87 (2)	1.99 (2)	2.8030 (17)	156 (2)
O2—H2*H*⋯O3^ii^	0.94 (2)	1.64 (2)	2.5780 (16)	178 (2)
N1—H1*N*⋯O4^iii^	0.97 (2)	1.80 (3)	2.763 (2)	170 (2)
N1—H2*N*⋯O2^iv^	0.90 (2)	2.44 (2)	2.9890 (18)	119.7 (16)
N1—H2*N*⋯O6^iv^	0.90 (2)	2.41 (2)	3.095 (2)	133.8 (17)
N1—H3*N*⋯O2^v^	0.88 (2)	2.50 (2)	2.9956 (19)	116.6 (15)
N1—H3*N*⋯O4^vi^	0.88 (2)	2.50 (2)	3.1125 (18)	127.5 (16)
N1—H3*N*⋯O5^vii^	0.88 (2)	2.48 (2)	3.181 (2)	137.3 (16)

Symmetry codes: (i) 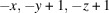 ; (ii) 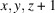 ; (iii) 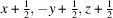 ; (iv) 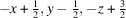 ; (v) 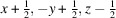 ; (vi) 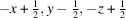 ; (vii) 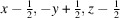 .

**Table 4:** Hydrogen-bond geometry (Å, °) for **III** [Chem scheme1]

*D*—H⋯*A*	*D*—H	H⋯*A*	*D*⋯*A*	*D*—H⋯*A*
O1—H1*H*⋯O2	0.85 (3)	2.32 (3)	2.746 (2)	112 (2)
O1—H1*H*⋯O3^i^	0.85 (3)	2.02 (3)	2.807 (2)	154 (3)
O2—H2*H*⋯O4^ii^	0.89 (3)	1.85 (3)	2.727 (2)	167 (3)
N1—H1*N*⋯O5^iii^	0.90 (3)	1.89 (3)	2.762 (2)	163 (2)
N1—H2*N*⋯O2^iv^	0.85 (3)	2.11 (3)	2.890 (2)	152 (2)
N1—H2*N*⋯O3^v^	0.85 (3)	2.49 (3)	2.974 (2)	117 (2)
N1—H3*N*⋯O4^vi^	0.89 (3)	2.00 (3)	2.776 (2)	146 (3)
N1—H3*N*⋯O5^vii^	0.89 (3)	2.61 (3)	3.112 (2)	117 (2)

Symmetry codes: (i) 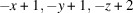 ; (ii) 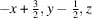 ; (iii) 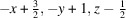 ; (iv) 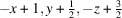 ; (v) 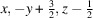 ; (vi) 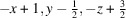 ; (vii) 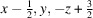 .

**Table 5:** Hydrogen-bond geometry (Å, °) for **IV** [Chem scheme1]

*D*—H⋯*A*	*D*—H	H⋯*A*	*D*⋯*A*	*D*—H⋯*A*
O1—H1*H*⋯O2	0.87 (1)	2.28 (3)	2.749 (3)	114 (3)
O1—H1*H*⋯O3^i^	0.87 (1)	1.95 (2)	2.754 (3)	152 (3)
O2—H2*H*⋯O1*W* ^ii^	0.88 (1)	1.84 (2)	2.693 (3)	164 (4)
N1—H1*N*⋯O4^iii^	0.86 (4)	1.99 (4)	2.850 (3)	178 (4)
N1—H2*N*⋯O5	0.86 (4)	1.91 (4)	2.766 (3)	172 (3)
N1—H3*N*⋯O3^iv^	0.86 (4)	2.27 (4)	2.927 (3)	133 (3)
N1—H3*N*⋯O6^v^	0.86 (4)	2.46 (4)	3.005 (3)	122 (3)
O6—H3*H*⋯O1*W* ^v^	0.88 (1)	1.83 (2)	2.686 (3)	164 (4)
O1*W*—H1*W*⋯O2^vi^	0.88 (1)	1.88 (1)	2.752 (3)	175 (4)
O1*W*—H2*W*⋯O4	0.88 (1)	1.82 (1)	2.698 (3)	177 (4)

Symmetry codes: (i) 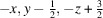 ; (ii) 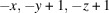 ; (iii) 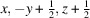 ; (iv) 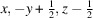 ; (v) 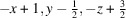 ; (vi) 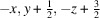 .

**Table 6:** Available crystal structures of dopamine forms

CSD refcode	Anion	Solvent	Comment	Reference
TIRZAX	None – free base structure	none	Zwitterion	Cruickshank *et al.* (2013 ▸)
DOPAMN01	Chloride	none		Giesecke (1980 ▸)
QQQAEJ02	Bromide	none		Pike & Dziura (2013 ▸)
ATOLUR04	Iodide	none	H-atom positions changed	Ivanova & Spiteller (2017 ▸)
CIZYAN	Nitrate	none	*Z*′ = 2	Gatfaoui *et al.* (2014 ▸)
OGEGAJ	Perchlorate	none		Boghaei *et al.* (2008 ▸)
ATOLIF	3-Carb­oxy-4-hy­droxy­benzene­sulfonate	1.5 H_2_O	H-atom positions changed	Ivanova & Spiteller (2010 ▸)
ATOMAY	Hydrogen squarate	none	H-atom positions changed	Ivanova & Spiteller (2010 ▸)
RAWDEB	Pyrene­tetra­sulfonate	5 H_2_O	Contains 1 dopamine and 3 guanidinium cations	Feng *et al.* (2017 ▸)
MIYLOV	3,5-Di­nitro­benzoate	none		Ohba & Ito (2002 ▸)
**I**	Benzoate	none		This work
**II**	4-Nitro­benzoate	none		This work
**III**	Ethane­disulfonate	none		This work
**IV**	4-Hy­droxy­benzene­sulfonate	1 H_2_O	Disordered anion	This work

**Table 7:** Selected hydrogen-bonding features in the structures of salt forms of dopamine The table shows the various potential acceptor (*A*) and donor (*D*) groups of the dopamine cation (first row) and details the types of fragment that form hydrogen bonds with these groups (body of table).

CSD refcode	Anion	*para*-OH *D*	*para*-OH *A*	*meta*-OH *D*	*meta*-OH *A*	1st NH *D*	2nd NH *D*	3rd NH *D*
DOPAMN01	Cl	anion	NH	anion		anion and OH	anion	anion
QQQARJ02	Br	anion	NH	anion		anion and OH	anion	anion
ATOLUR04	I	anion	NH	anion	NH	anion and OH	anion and OH	anion
CIZYAN Fragment 1	NO_3_	2 O of anion		anion	NH	anion and OH	2 O of anion	2 × anions
CIZYAN Fragment 2	NO_3_	anion	NH	anion	NH	OH	2 O of anion	2 × anions and OH
OGEGAJ	ClO_4_	anion	NH	anion	NH	anion and OH	anion	OH
ATOLIF	carb­oxy­hydroxy­benzene­sulfonate	anion	H_2_O	H_2_O		anion	anion	H_2_O
ATOMAY	squarate	anion	2 × NH	anion		anion	anion	2 × OH
RAWDEB	pyrene­tetra­sulfonate	H_2_O		anion	2 × NH (guad)	H_2_O	H_2_O	H_2_O
MIYLOV	di­nitro­benzoate	anion		anion (nitro)		anion	anion (nitro)	2 O of anion
**I**	benzoate	anion		anion		anion	anion	anion
**II**	nitro­benzoate	2 O of anion		anion		anion	anion (nitro)	2 × anion (COO and nitro)
**III**	ethane­disulfonate	anion	NH	anion		anion	anion	OH
**IV**	hy­droxy­benzene­sulfonate	H_2_O	H_2_O	anion		anion and OH	anion	anion

**Table 8:** Groups identified as having isostructural packing of cations

	Group 1	Group 2	Group 3	Group 4	Group 5	Group 6
Cations	Dop only	Dop and Tyr	Dop and Tyr	Tyr only	Tyr only	Tyr only
Structure	ATOLUR04 [dop][I]	**I** [dop][benzoate]	DOPAMN01 [dop][Cl]	MEDGEJ	MEDBAA	MEDBOO
	OGEGAJ [dop][ClO_4_]	MEDDEG [tyr][4-amino­benzoate]	QQQAEJ02 [dop][Br]	MEDGUZ	MEDBII	MEDBUU
		MEDFEI [tyr][4-methyl­benzoate]	**III** [dop][ethane­disulfonate]	MEDHEK		MEDCEF
			TIRZEB Tyramine hemihydrate			MEDDOQ
			TYRAMC11 [tyr][Cl]			
			MECYAW [tyr][Br]			
			MECYIE [tyr][BF_4_]·H_2_O			
			MECYUQ [tyr][PO_4_H_2_]·2H_2_O			
			MECYOK [tyr][ClO_4_]·H_2_O			
Comments		Mixed cations with benzoate or *para*-substituted benzoate anions	Mixed cations and neutral tryamine; mixed anhydrate and hydrate; simple inorganic anions	All di­carboxyl­ate anions	Benzoate and halobenzoate anions	All halo or methyl-substituted benzoate anions

Note: Dop is the dopamine cation and Tyr is the tyramine cation.
